# Nanobody-Based Theranostic Agents for HER2-Positive Breast Cancer: Radiolabeling Strategies

**DOI:** 10.3390/ijms221910745

**Published:** 2021-10-04

**Authors:** Ivanna Hrynchak, Liliana Santos, Amílcar Falcão, Célia M. Gomes, Antero J. Abrunhosa

**Affiliations:** 1 ICNAS-Produção Unipessoal, Lda.—University of Coimbra, 3000-548 Coimbra, Portugal; ivanna.ua@icnas.uc.pt (I.H.); liliana.santos.ca26@gmail.com (L.S.); 2 CIBIT/ICNAS—Institute for Nuclear Sciences Applied to Health, University of Coimbra, 3000-548 Coimbra, Portugal; amilcar.falcao@uc.pt; 3 iCBR—Coimbra Institute for Clinical and Biomedical Research, Faculty of Medicine, University of Coimbra, 3000-548 Coimbra, Portugal; cgomes@fmed.uc.pt; 4 CIBB—Center for Innovative Biomedicine and Biotechnology, University of Coimbra, 3000-548 Coimbra, Portugal; 5 CACC—Clinical Academic Center of Coimbra, 3000-075 Coimbra, Portugal

**Keywords:** nanobodies, targeted radionuclide therapy, HER2 breast cancer, radiolabeling strategies, nuclear imaging

## Abstract

The overexpression of human epidermal growth factor 2 (HER2) in breast cancer (BC) has been associated with a more aggressive tumor subtype, poorer prognosis and shorter overall survival. In this context, the development of HER2-targeted radiotracers is crucial to provide a non-invasive assessment of HER2 expression to select patients for HER2-targeted therapies, monitor response and identify those who become resistant. Antibodies represent ideal candidates for this purpose, as they provide high contrast images for diagnosis and low toxicity in the therapeutic setting. Of those, nanobodies (Nb) are of particular interest considering their favorable kinetics, crossing of relevant biological membranes and intratumoral distribution. The purpose of this review is to highlight the unique characteristics and advantages of Nb-based radiotracers in BC imaging and therapy. Additionally, radiolabeling methods for Nb including direct labeling, indirect labeling via prosthetic group and indirect labeling via complexation will be discussed, reporting advantages and drawbacks. Furthermore, the preclinical to clinical translation of radiolabeled Nbs as promising theranostic agents will be reported.

## 1. Introduction

Breast cancer (BC) is the second leading cause of mortality for women worldwide [1]. BC harboring overexpression of the receptor tyrosine kinase human epidermal growth factor receptor 2 (HER2) and/or amplification of the HER2/neu gene accounts for about 20% of all BCs [2]. Furthermore, HER2 expression can change during the course of the disease and can be unequally expressed across primary tumor and metastatic lesions. Excessive HER2 signaling triggers activation of downstream pathways, promoting proliferation, motility and survival rate of cancer cells, which ultimately translates into an aggressive behavior, with a higher risk of metastases and shorter overall survival [3,4]. In light of its key biological role in tumorigenesis, HER2 has become an attractive target for BC diagnosis and therapy.

Since only a subset of BC patients has HER2-positive (HER2^+^) tumors, robust assessment of HER2 expression represents a critical step in selecting patients who might benefit from HER2-targeted therapies. Currently, HER2 status is determined by immunohistochemistry (IHC) and fluorescence in situ hybridization (FISH), both requiring invasive procedures for biopsy sample collection [5]. These histopathological methods do not address the whole-tumor heterogeneity and are limited by the sampling of a site at a certain time point, producing many times false-negative or false-positive results, which further impact on the proper selection of treatments [6,7]. Moreover, these techniques are not informative about differences in HER2 levels between primary HER2^+^ BC and distant metastases [8,9,10,11]. Therefore, a more comprehensive method for the assessment of HER2 expression in both primary tumors and distant metastasis is needed. Molecular imaging techniques using radiopharmaceuticals can be used to reduce this source of incertitude in the evaluation of HER2 expression. This non-invasive imaging approach has the potential to provide information about the global status of HER2 in primary and distant metastatic lesions, at the same time [12]. Furthermore, this approach allows us to monitor the response to HER2-targeted therapies and to identify the patients who become resistant [12,13,14].

The development of HER2-targeted therapies using monoclonal antibodies (mAb), such as trastuzumab and pertuzumab and tyrosine kinase inhibitors, such as lapatinib, has significantly improved the survival of patients with HER2^+^ BC. However, HER2-targeted therapy remains a challenge since not all patients respond and a significant number of the responders eventually relapse or become resistant to the therapy [15]. Further efforts are still needed toward designing more specific and effective therapeutic agents for treating patients resistant to HER2-targeted therapies. One alternative strategy is targeted radionuclide therapy (TRNT) in which a tumor-specific molecule labeled with a cytotoxic radionuclide is used to locally irradiate targeted tumor cells.

Several HER2-targeted vehicles, such as monoclonal antibodies (mAb), antibody-based fragments (Fab), diabodies, minibodies, nanobodies (Nb) and affibodies, have been explored for HER2^+^ BC imaging and TRNT in the past few years [16]. In particular, Nb, also referred to as single-domain antibodies or VHH molecules, are proteins based on the smallest functional fragments of heavy chain antibodies occurring in *Camelidae* [17] with attractive features for radiolabeled imaging and TRNT applications [18]. The optimal choice of radiolabeling strategies will determine the Nb potential as imaging agents and therapeutics, that are dependent on their interaction with targeted cells.

In this review, we will provide a general overview of antibody-based molecular imaging, with a special focus on Nb as imaging tracers and vehicles for TRNT. Subsequently, we will provide the current knowledge regarding the radiolabeling strategies of Nb and the challenges facing the translation of preclinic studies into the clinical setting.

## 2. Nuclear Imaging and Targeted Radionuclide Therapy

Nuclear imaging has been extensively used in the detection, diagnosis and staging of BC and offers several advantages in the context of the clinical practice as well as in clinical and preclinical research [19,20]. Nuclear imaging uses trace amounts of radiolabeled molecules—radiopharmaceuticals—allowing the non-invasive real-time visualization of biochemical processes at the cellular and molecular levels in living subjects [21,22,23]. Radiopharmaceuticals are usually administered systemically and are intended to accumulate in their target sites for diagnostic imaging or targeted therapy. Patients’ whole-body images are acquired in dedicated nuclear medicine tomographic cameras to detect gamma-photons emitted from sites of radiopharmaceutical accumulation, which can be further quantified [20].

Positron emission tomography (PET) and single-photon emission computed tomography (SPECT) are routinely used in clinical oncology in the diagnosis and follow-up of cancer patients. SPECT imaging relies on the use of radionuclides that emit single gamma (*γ*)-ray photons with different energies and varying half-lives such as technetium-99 (^99m^Tc; Eγ = 140 keV, t_1/2_ = 6 h), indium-111 (^111^In, Eγ = 245 keV, t_1/2_ = 2.8 days), or iodine-123 (^123^I, Eγ = 159 keV, t_1/2_ = 13.2 h), while PET makes use of tracers labelled with radioisotopes that decay by emission of a positron (β^+^ particle), such as fluorine-18 (^18^F; Eβ^+^ = 634 keV, t_1/2_ = 1.8 h), copper-64 (^64^Cu, Eβ^+^ = 653 keV, t_1/2_ = 12.7 h), gallium-68 (^68^Ga, Eβ^+^ = 1899 keV, t_1/2_ = 1.13 h), yttrium-86 (^86^Y, Eβ^+^ = 3150 keV, t_1/2_ = 14.7 h) and zirconium-89 (^89^Zr, Eβ^β+^ = 901 keV, t_1/2_ = 78.4 h) [23,24,25,26,27]. The positron after interacting with nearby-electron produce two annihilation gamma photons of 511keV emitted in opposite directions generating high-quality images with increased sensitivity and spatial resolution, compared to single-photon emission tomography [27,28]. For instance, PET images are able to detect mammographically occult lesions, as well as distant metastases mainly in patients with radiographically dense breasts and poor differentiation of recurrent disease and scar tissue following surgery and/or radiotherapy [29].

Over the last few years, nuclear imaging experienced substantial developments and innovation in radiopharmaceuticals and nowadays has a clearly defined role in clinical oncology for patient prognosis, risk-stratification, staging, molecular-based diagnosis, pre-therapeutic dosimetry, monitoring of treatment response and early detection of residual disease [30,31]. This information is essential in the tailoring of medical treatment to each patient, allowing for the optimization of the therapeutic regimen, minimizing the risks of toxicity as well as reducing cost and patient distress [30,31].

The nano-to-picomolar sensitivity of PET with the specificity and affinity of antibodies for a particular target, extended the PET applications into the field of immuno-oncology, providing a powerful tool to implement the concept of personalized medicine. Immuno-PET imaging has proved excellent specificity and sensitivity in detecting cancer-specific antigens (e.g., HER2), being considered a potential complement of IHC staining in clinical dilemmas when suspected lesions are inaccessible for biopsy. In addition to improving triage during early disease stages, it also facilitates image-guided surgery providing a non-invasive and quantitative assessment of tumor target expression and distribution [32]. These advantages led to advances in bioconjugation strategies for developing radiolabeled full-size or antibody fragments for medical applications.

In particular, ^89^Zr-labeled trastuzumab (Herceptin^®^), one the most widely used mAb in clinical oncology, has been used to evaluate the HER2 status for patient selection and to assess the response of BC patients to anti-HER2 therapy [33,34] and represents an encouraging step towards the routine use of PET to accurately assess the expression of HER2 over time. Meanwhile, HER2-targeted PET imaging using ^64^Cu-NOTA-Trastuzumab has been reported as robust immune-PET agents for BC patients [35,36].

Apart from diagnosis, antibodies can be used as platforms for TRNT, representing a rapidly expanding group of effective anti-cancer drugs. Radioisotopes commonly used emit alpha- (*α*) or beta- (*β*^−^) particles, which cause DNA damage through reactive oxygen species production, single and double DNA stranded breaks and inhibition of DNA repair mechanism. The most commonly exploited for therapeutic purposes include iodine-131 (^131^I, *β*^−^) lutetium-177 (^177^Lu, *β*^−^), yttrium-90, (^90^Y, *β*^−^), actinium-225 (^225^Ac, *α*) and astatine-211 (^211^At, *α*).

The exceptional target specificity and affinity of antibodies for tumor-associated antigens provide a unique means to efficiently deliver ionizing radiation to a disseminated tumor and small metastasis while sparing the surrounding normal tissues and reducing adverse events [18,37,38]. Several mAb or antibody-related therapeutics have been approved by the US Food and Drug Administration (FDA) and the European Medicines Agency (EMA) for use in cancer patients, being trastuzumab the most frequently investigated therapeutic mAb in molecular imaging [39].

An important factor for treatment success is the quantitative assessment of biodistribution and tumor uptake before therapy. In this respect, an antibody-based theranostic approach coupling imaging and therapy designed for the same target provides insights into tumor heterogeneity, proof of access to tumor lesions and prediction of treatment outcome. Such an approach represents a powerful tool in the development of personalized antibody-based therapies and is currently being investigated across numerous centers.

## 3. Antibody Fragments for Nuclear Imaging and Radionuclide-Based Therapy

Full-length antibodies are of particular interest in nuclear medicine due to their well-defined structure, relative stability and high specificity and affinity for target antigens. These features make them strong candidates for targeted molecular imaging and TRNT [38]. The intact immunoglobulin (IgG;150 kDa) is a multimeric binding protein composed of antigen-binding (Fab) domains and a constant region (Fc) that interacts with cell-surface receptors. The Fc also interacts with the neonatal Fc receptor (FcRn), which is involved in antibody recycling and maintenance in circulation. The use of radiolabeled intact mAbs for imaging and therapy remains challenging because of their long circulating half-life (days to weeks) due to their size and FcRn interaction. The slow clearance of mAbs from the blood results in high background and low target-to-background ratio, whereby good contrast images can only be obtained hours or days after injection [40,41]. Therefore, long-lived radionuclides are required for radiolabeling mAbs, which causes a substantial high dose burden to patients [42]. In addition to that, the high binding affinity of intact mAbs limits tumor penetration and leads to heterogeneity of intra-tumor distribution, as the antibodies can get trapped at the tumor periphery [43]. This partial tumor penetration can subsequently result in an untargeted subpopulation of cancer cells, resulting in reduced efficacy of antibody-based therapy and the emergence of resistant subclones [44]. Furthermore, other factors such as the antigen density, extravascular binding of mAbs, vascularization, capillary permeability, tissue structure and composition and interstitial pressure can also lead to a heterogeneous distribution of mAbs within the tumors [16,38]. Moreover, mAbs also present unspecific uptake on target-negative sites due to tumor enhanced permeability and retention (EPR) effect [45], which increases the risk of false-positive results [33]. Another disadvantage is the inability of mAbs to cross the blood–brain barrier, which limits their application to central nervous system malignancies [46]. To overcome these functional drawbacks, several smaller antibody fragments with different molecular weights have been generated, which are summarized in Table 1.

Antibody fragments are engineered parts of antibodies that maintain the desired high affinities and specificities of full-intact antibodies, but with more compatible pharmacokinetics for radionuclide molecular imaging or TRNT [38]. Compared to intact IgG, smaller fragments have shorter circulation times, deeper tumor penetration and high specificity to the target, resulting in high tumor-to-normal tissue ratios and high contrast images at earlier times. The absence of the Fc region is an advantage since it reduces the nonspecific binding of the fragment to other cells and eliminates FcRn recycling, improving image quality. For therapeutic applications, antibody fragments allow rapid tumor uptake and elimination, which limits radiation exposure to normal tissues and increases therapeutic indexes [50].

Antibody fragments can be produced through either enzymatic digestion or genetic engineering. The enzymatic digestion of antibodies with the enzymes pepsin or papain results in the smaller fragments Fab (55 kDa) and (Fab’)2 (110 kDa), with one or two antigen-binding regions, respectively, but lack of the Fc region. These fragments offer better contrast in comparison with intact mAb reducing the time between injection and imaging acquisition. However, these fragments are still too large to achieve efficient extravasation and display a decrease in the apparent binding affinity when compared to its whole mAb precursor [47].

Great effort has been devoted to the development of antibody-based imaging probes, namely single-chain variable fragments (scFv). scFvs are fusion proteins formed by joining the variable regions of an antibody’s heavy (VH) and light (VL) chains with a versatile linker peptide. These fragments have a molecular weight of 25 kDa and their monovalent structure has a subnanomolar affinity for a single antigen. Despite the good imaging contrast provided by radiolabeled scFvs, the tumor uptake remains low because of the suboptimal ratio between the blood clearance and extravasation rates. Engineering of the dimeric bivalent form of scFv fragments (diabody, ~50 kDa) and/or fusions of the scFv to the Fc region of IgG (minibody, ~80 kDa) have resulted in higher tumor accumulation and good imaging contrast within few hours post-injection. However, these antibody-based fragments are prone to denaturation, spontaneous dimerization and the formation of immunogenic aggregates [51].

Alternative engineered scaffold proteins are being successfully employed as targeting imaging tracers, due to their high affinity and specificity. A widely explored scaffold are affibodies, a class of small proteins (6–7 kDa) scaffolds composed of 58 amino acid sequences folded into three alpha-helices. They are ideal candidates for acting as imaging probes due to their small size, chemical robustness and high affinity in the nanomolar range [52]. HER2-targeted affibody molecules have demonstrated a high potential for radionuclide molecular imaging in preclinical settings [48,53,54,55]. Clinical studies using the anti-HER2 affibodies ABY-002 [56] and ABY-025 [49,57] have provided a high contrast specific imaging of HER2^+^ lesions when labeled with ^111^In or ^68^Ga. However, the development of radiolabeled affibodies is costly and presents some difficulties in terms of scaling up the manufacturing process [58]. Furthermore, the labeling methods can result in increased lipophilicity, which often results in off-target interactions with normal tissue and binding to blood proteins [59].

One of the most promising approaches for the development of antibody-based imaging probes is the use of single-domain antibodies or nanobodies (Nbs), which are the smallest naturally occurring antigen-binding fragments, derived from camelid and shark immunoglobulins. In addition to the usual mAb existing in the serum, camelids were found to produce atypical antibodies devoided of light chains, called ‘heavy-chain-only antibodies’ (HcAbs). These HcAbs comprise two constant domains (C_H_2 and C_H_3), a hinge region and an antigen-binding or variable heavy chain domain (V_HH_) called the Nb, which retains full antigen-binding ability [60]. Nbs have a small size (4 nm long and 2.5 nm wide), low molecular weight (~15 kDa), low immunogenicity, high solubility, stability, specificity and affinity, which enables a rapid tumor accumulation with a homogeneous distribution [61]. The monomeric structure of Nbs and the lack of post-translational modifications allow for their recombinant production in microorganisms, which is easy and cost-effective [62]. Due to their low molecular weight, Nbs are excreted by the kidney leading to a very short biological half-life and offering high contrast images immediately after administration, which are very desired properties for imaging [63]. Additionaly, a recent animal study confirmed that the Nbs extravasate faster than mAbs and are distributed uniformly inside the tumor, resulting in better signal-to-noise and less toxic effects [64]. Despite, the rapid clearance of Nbs through the urinary tract, the biodistribution is antigen-specific, resulting in a high tumor to background ratio early after administration, thus facilitating diagnostic scans. Moreover, Nbs have uniquely advantageous properties such as prolonged shelf-life at 4 °C and −20 °C, tolerance to increased temperature (60–90 °C, several weeks at 37 °C), resistance to proteolytic degradation, stability at non-physiological pH (range 3.0–9.0) and elevated pressure (500–750 MPa) and chemical denaturants (2–3 M guanidinium chloride, 6–8 M urea), while preserving their antigen-binding capability [65,66,67]. These characteristics make them desirable for imaging agents as they open more possibilities for conjugation chemistry to chelators, contrast agents, or optical probes. In addition, they are sufficiently stable to be administrated by intravenous, oral, intraperitoneal, or intratumoral routes. A first-in-human study demonstrated the safety and tumor-targeting potential of ^68^Ga-radiolabeled Nbs directed against HER2 in breast cancer patients in both primary lesions and metastasis [68]. A recent preclinical study demonstrated that radiolabeled Nbs, in contrast to mAbs, were able to cross the blood-brain barrier and might represent promising vehicles for molecular imaging and targeted radionuclide therapy for brain metastatic lesions [69]. Currently, a phase II clinical trial using the ^68^Ga-NOTA anti-HER2 Nbs to detect brain metastasis in BC patients is ongoing (NCT03331601).

Regarding TRNT, although the small size of a monomeric Nb is beneficial in molecular imaging, it can be a disadvantage for therapy as Nbs might be quickly eliminated through the kidneys since their molecular weight is well below the 50–60 kDa renal threshold for glomerular filtration. Therefore, due to their rapid clearance, only a marginal fraction of the administered Nb will reach its cognate target, limiting their therapeutic efficacy. Thus, monomeric Nb should be frequently administered to obtain a high target load in vivo, or alternatively, half-life extension should be considered by the construction of multimeric Nb or by fusion of Nbs to serum albumin (directly or via an albumin-binding Nb) [70].

## 4. Radiolabeling Methods of Nanobodies

Non-invasive tracking of Nbs by PET/SPECT requires the incorporation of a suitable radionuclide into the structure without interfering with affinity and specificity [26]. While some radionuclides, such as ^124^I and ^131^I, can be coupled directly to Nbs, others such as ^18^F and ^89^Zr require indirect labeling methods, using prosthetic groups or bifunctional chelators. Radiolabeling strategies depend primarily on the radionuclide used [71] and on the Nbs fragment available for conjugation. The radionuclide needs to be covalently bound to the fragment and to remain kinetically and thermodynamically stable over the course of the study.

Radiolabeling options for smaller antibody fragments like scFv, diabodies, affibodies and Nbs are more limited than for intact antibodies since they are smaller and have fewer available sites for conjugation. In intact antibodies, the cysteine residues that form the inter-chain disulfide bonds can be used for specific conjugation sites, an approach not possible in Nbs due to the missing inter-chain disulfide bridges. In addition, the site-specific conjugation with modified glycans used for intact antibodies is not possible for smaller fragments since the C_H_2 domain region is no longer present.

However, free cysteine residues can be engineered into small fragments to provide free thiol groups for further conjugation in predetermined specific sites [58]. These emerging site-specific bioconjugation strategies provide homogenous tracer populations in terms of immunoreactivity and pharmacokinetic behavior. The positioning of the label opposite to the antigen-binding site avoids the risk of interference with the binding capacity of Nbs [72].

Most common methods to label smaller fragments exploit either the reactive primary amine groups on lysine residues or the thiol groups of cysteines [58,73]. However, this random process results in a heterogeneous mixture, relying on the particular protein sequence of the employed Nbs. This imposes a big challenge not only for consistent manufacturing but also blocks straightforward characterization, due to this lack of control on the number of labels per Nb and the exact location of the probe attachment [72].

**Figure 1:**
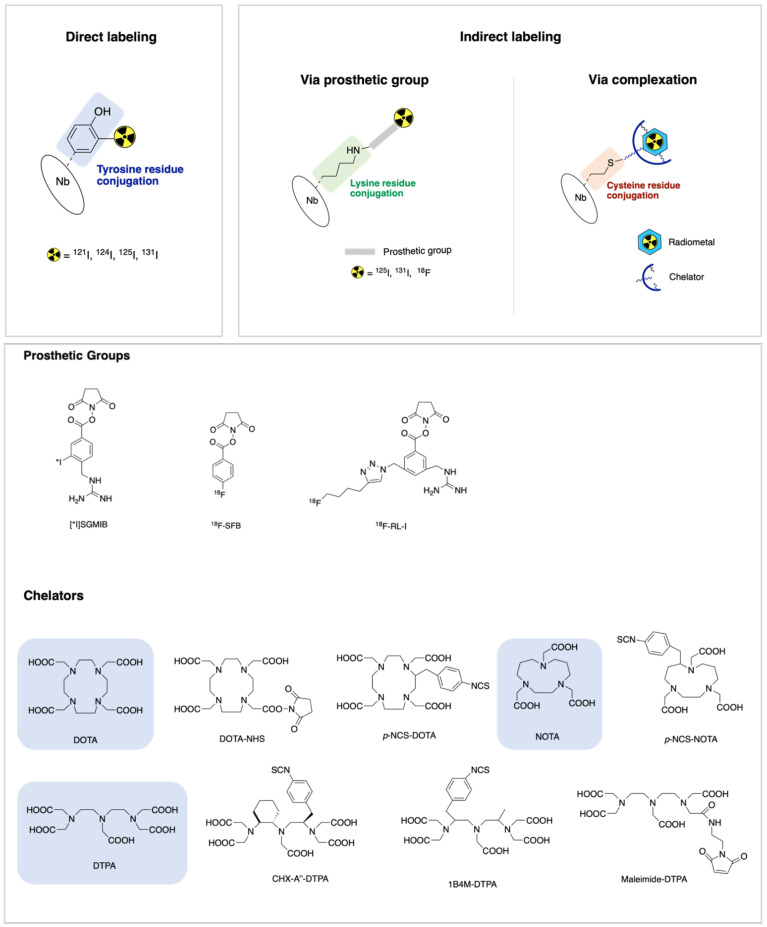
Illustration of three main strategies for the radiolabeling of Nbs: direct labeling, indirect labeling via a prosthetic group and indirect labeling via complexation. In the bottom part, the most commonly used prosthetic groups, macrocyclic and acyclic chelator for indirect radiolabeling via prosthetic group and complexation, respectively, are depicted.

There are three main strategies for radiolabeling Nbs: direct labeling, indirect labeling via a prosthetic group and indirect labeling via complexation (Figure 1) [71]. The principles of each method will be addressed in the following sections. Table 2 summarizes the most frequently used radiolabeling strategies for Nb targeting HER2^+^ BC.

### 4.1. Direct Labeling

The radioactive isotopes of iodine (^123^I, ^124^I, ^125^I and ^131^I) can be directly integrated into a Nb molecule by electrophilic substitution at tyrosine and histidine residues. The first step consists of creating the electrophile *I^+^ using oxidizing agents like chloramine T (Iodogen^®®^) and *N*-halosuccinimides. Next, the electrophile attacks the aromatic ring of the amino acid tyrosine, since it contains the electron-donating hydroxyl group, forming and stabilizing the σ-complex. This attachment of iodine to tyrosine is highly suitable because the labeling takes place under mild conditions [71]. A similar approach was performed by Pruszynski et al. [74], using 5F7GGC Nb radioiodinated with ^125^I via Iodogen^®®^ in a radiochemical yield (RCY) of 83.6 ± 5.0%. However, in the same study, better tumor-targeting properties were obtained by radioiodination of the same Nb using [^131^I]IB-Mal-D-GEEEK as a prosthetic group. Therefore, some disadvantages have to be taken into consideration on this direct strategy. This radioiodination method is only possible when the protein contains accessible tyrosine or histidine residues. Moreover, an increasing in vivo toxicity can occur due to the accumulation of iodine in the thyroid and stomach.

Similar to iodination, the positron emitter ^18^F can also be directly incorporated into a Nb through a tyrosine residue. In theory, this could be achieved via electrophilic or nucleophilic substitution but, in practice, the nucleophilic fluorination approach is the most routinely used. As harsh conditions are required for nucleophilic substitution, this approach is not suitable for the radiolabeling of proteins [71].

Small antibody fragments can also be radiolabeled via an indirect approach, using a prosthetic group or a bifunctional chelator complex. Both strategies will be discussed in the next subsections.

### 4.2. Indirect Labeling via Prosthetic Group

In this approach, indirect halogenation such as iodination and fluorination occurs through the incorporation of a bifunctional prosthetic group enabling both radiolabeling and binding to the protein [71]. The importance of using a residualized prosthetic group for radiolabeling Nbs has been described in previous publications that demonstrated increased intracellular retention and in vivo tumor uptake [74,75].

One of the prosthetic groups evaluated, *N*-succinimidyl-3-guanidinomethyl-5-[^131^I]iodobenzbate (SGMIB, Figure 1) has many of the desired properties for TRNT including enhanced tumor uptake, in vivo stability and rapid clearance from normal tissues, including the kidneys. It has been used to synthesize *iso*-[^221^At]SGMIB, [^221^At]SGMIB and their radioiodinated analogs *iso*-[^131^I]SGMIB) and [^131^I]SGMIB to react with the anti-HER2 Nb 5F7. Preclinical studies with [^211^At]SAGMB-5F7 demonstrated high and prolonged tumor targeting and rapid normal tissue clearance, with even more favorable observed with *iso*-[^211^At]SAGMB-5F7. Similarly, *iso*-[^131^I]SGMIB-5F7 was shown to offer significantly improved tumor targeting compared with [^131^I]SGMIB-5F7 [77].

In the same year, another anti-HER2 Nb, 2Rs15d, was radiolabeled with ^131^I via the residualizing prosthetic group [^131^I]SGMIB with promising results: [^131^I]SGMIB-2Rs15d showed a low toxicity profile and significant therapeutic efficacy. The goal of this study was to generate a more potentially theranostic drug than 5F7 Nb since it competes with trastuzumab for binding to domain IV on HER2 and does not offer solutions to HER2 treatment resistance mechanisms [78].

In a more recent study, Feng and co-workers [79] evaluated a site-specific strategy using maleimide moiety—MEGMIB—as a novel residualizing prosthetic agent for radioiodonation and an anti-HER2 Nb—5F7GGC—with GGC tail, for conjugation via Michael addition. This tracer was designed to contain the *iso*-SGMIB motif but with the *N*-hydroxysuccinimide (NHS) ester replaced by a maleimide moiety for thiol conjugation, thus obtaining [^131^I]MEGMIB-5F7GGC. This was later compared with the previous lead agent *iso*-[^125^I]SGMIB-5F7 and was expected a different in vivo behavior of proteins modified via maleimide-thiol Michael addition reaction from the same protein modified with an active NHS ester. The site-specific conjugate [^131^I]MEGMIB-5F7GGC exhibited similar binding affinity, immunoreactivity and intracellular trapping capacity but better homogeneity. Still, it was observed that in mice with HER2-expressing SKOV-3 xenografts [^131^I]MEGMIB-5F7GGC has tumor targeting and retention similar to *iso*-[^125^I]SGMIB-5F7, but had significantly lower uptake in kidney and other normal tissues.

Regarding ^18^F, *N*-succinimidyl 4-[^18^F]fluorobenzoate ([^18^F]-SFB, Figure 1) is the most frequently used prosthetic group for the fluorination of Nbs. This activated ester was conjugated to the ε-amino group of lysine residues of 2Rs15d Nb via acylation reaction. The ^18^F labeled Nbs were obtained in good conjugation yield (20–30%) and showed excellent targeting properties and specificity for HER2. Furthermore, this tracer was also evaluated in vivo and [^18^F]SFB-2Rs15d did not compete with trastuzumab, which indicates that this tracer could be used for diagnosis or staging of patients undergoing trastuzumab therapy [80]. Several other approaches of radiolabeling with ^18^F have been successfully applied [89].

A novel residualizing prosthetic group, ^18^F-RL-I (Figure 1), synthesized by Vaidyanathan and co-workers, was used to label the 5F7 Nb whilst preserving immunoreactivity (62–80%) and affinity (4.7 ± 0.9 nM) for HER2 [81]. Later, the tumor-targeting potential of the 5F7 anti-HER2 Nb labeled with ^18^F-RL-I and ^18^F-SFB was evaluated. Higher tumor uptake was obtained when the Nb was labeled with ^18^F using the [^18^F]RL-I compared with [^18^F]SFB. Considerably higher renal uptake was also seen with this new prosthetic agent, which means that both ^18^F-RL-I-5F7 and ^18^F-SFB- 5F7 require further evaluation as tracers for the evaluation of HER2-expressing cancers using immuno-PET [76]. Although several studies have shown that the tumor uptake of two ^18^F-labeled Nbs—2Rs15d and 5F7—that target internalizing receptors, such as HER2, can be augmented by using residualizing prosthetic agents, this strategy resulted in undesirable effects such as a very high uptake in the kidneys [76,90,91,92]. Another disadvantage of using prosthetic groups to incorporate ^18^F is that the formation of prosthetic groups often requires multistep complex methods, resulting in decreased yields [71].

### 4.3. Indirect Labeling via Complexation

This method includes the incorporation of metallic radioisotopes into an Nb using a chelator. Factors such as imaging modality (PET or SPECT), match of the half-life of the radioisotope to the pharmacokinetics of the Nb and the availability of the radioisotope influence the choice of the radiometal. After conjugation, the radiometal-chelator complex must be highly thermodynamically and kinetically stable, which is essential to ensure that the radiometal remains bound to the Nb in vivo [93]. The most widely used chelators include diethylene triamine pentaacetic acid (DTPA), 1,4,7,10-tetraazacyclododecane-1,4,7,10-tetracetic acid (DOTA) and 1,4,7-triazacyclononane-1,4,7-triacetic acid (NOTA) (Figure 1). The covalent attaching of these chelators to Nbs—conjugation—is performed via reactive electrophilic groups such as *N*-hydroxysuccinimide esters (NHS), isothiocyanates (SCN) and anhydrides that react with the ε-amino group of lysines on the Nb at mild conditions [93,94,95]. However, the presence of multiple amino acids in a Nb structure may lead to a lack of site-specificity, resulting in heterogeneous mixtures of chelator-protein conjugates. This random conjugation can exhibit suboptimal pharmacokinetics and decreased affinity for target receptors [93,94]. In this sense, strategies for site-specific labeling of Nbs have been developed. An example of a random and site-specific strategy is described below.

In a study performed by Xavier et al., a new anti-HER2 tracer—^68^Ga-NOTA-2Rs15d—was successfully developed via a rapid procedure under mild conditions (RCY of >97% and with a specific activity of 55–200 MBq/nmol). The 2Rs15dHis_6_ Nb was selected for the absence of hexahistidine (His_6_) tag in its antigen-binding loops, leading to a successful conjugation of a *S*-2-(4-isothiocya-natobenzyl)-NOTA (*p*-SCN-Bn-NOTA) to lysines and posteriorly radiolabeled with ^68^Ga without compromising the antigen-binding capacity. For this study, ^68^Ga (t_1/2_ = 1.13 h) was the positron-emitter chose in accordance with the short biological half-life of this Nb. In addition, the macrocyclic chelator NOTA is interesting for ^68^Ga complexation due to their fast and efficient radiolabeling and high in vivo stability (log K_ML_ = 31.0). However, this random labeling leads to a heterogeneous mixture of Nb conjugated to NOTA chelator. Results demonstrated that this new tracer showed specific accumulation in xenografts in ex vivo biodistribution studies and PET/CT imaging. Moreover, the removal of the His_6_ tag considerably reduced kidney retention of the Nb by 60% [82]. Pre-clinical evaluation of this radiolabeled anti-HER2 Nb revealed a good toxicological profile and a low radiation burden, enabling the construct to enter phase I clinical trials on humans. The results scored well in terms of efficiency, tracer accumulation and safety as no adverse effects were detected, rendering this construct suitable to enter phase II clinical trials [68]. His_6_ tag of the Nb can be directly labeled with ^99m^Tc (CO)^3^ without any chemical modification of the protein, as shown for an anti-HER2 Nb [83].

A generic strategy for the site-specific labeling of Nbs via thioether bond was reported by Massa et al. [88]. An unpaired cysteine was introduced at the carboxyl-terminal end of the Nb to eliminate the risk of antigen-binding interference, which required a reduction step before conjugation because of the spontaneous dimerization. This reduced probe was subsequently conjugated to maleimide-DTPA, for labeling with ^111^In (t_1/2_ = 2.83 h), resulting in the production of a homogeneous group of the tracer with a specific activity of 9–49 MBq/nmol.

There are two ways in which the connection between radiometal and the vector can be established: pre-labeling and pos-labeling. In the first one, the radiometal is complexed with a bifunctional chelator prior to the interaction with the vector molecule, while in the pos-labeling strategy, the bifunctional chelator is first connected to the Nb, followed by radiometal complexation. The latter strategy is most commonly used as the chelator-Nb conjugate can be stored in large quantities and subsequently used in small aliquots for radiolabeling [85].

D’Huyvetter et al., shown that the choice of the bifunctional chelator has an important effect on the behavior of the radiolabeled conjugates. For the development of a Nb-based radiopharmaceutical, four chelating agents DOTA–NHS–ester, *p*-SCN–Bn–DOTA, CHX-A″–DTPA and 1B4M–DTPA (Table 3, entry 6–9) were tested and the optimal chelator for ^177^Lu complexation was 1B4M–DTPA. Their favorable characteristics exhibit better biodistribution: significantly higher kidney uptake was reported for the DOTA-based conjugates compared to the DTPA-based conjugates of anti-HER2 Nb, despite being predominant and persistent [85]. To optimize the ^177^Lu-DTPA-2Rs15d tracer for reduced kidney retention, three 2Rs15d Nbs were produced with different C-terminal amino-acid tag sequences (Myc-His-tagged, His-tagged and untagged) and the study showed that untagged ^177^Lu-DTPA-anti-HER2 Nb (Table 3, entry 10) almost completely blocked tumor growth in xenograft mouse models bearing small tumors, coinciding with increased event-free survival. This means that the amino acid composition and polarity at the *C*-terminus affect kidney retention [86]. For the success of future therapeutic Nbs-based applications, it is crucial to reduce kidney retention of radiolabeled Nbs as this could lead to renal toxicity.

Recently, the 2Rs15d Nb was labeled with a therapeutic α-emitter, ^225^Ac, via DOTA chelation with a high yield (<90%) and radiochemical purity (RCP) above 95% (Table 3, entry 11). Before, DOTA was conjugated to the Nb through the ε-amino groups of the lysines, forming a stable thiourea bond. Ex vivo biodistribution of [^225^Ac]-2Rs15d in SKOV3 tumor-bearing mice showed high tumor uptake and exceptionally low kidney retention when co-injected with the plasma expander Gelofusin. Additionally, in in vitro studies, [^225^Ac]-2Rs15d demonstrated excellent preservation of immunoreactivity and affinity for its HER2 target [87].

A study in 2020 by Puttemans and co-workers evaluated the anti-HER2 Nb 2Rs15d, coupled to diagnostic *γ^−^* and therapeutic *α^−^* and *β^−^* emitting radionuclides for the detection and treatment of HER2 brain lesions in a preclinical setting. For this purpose, the Nbs 2Rs15d radiolabeled with ^111^In, ^225^Ac and ^131^I using DTPA- and DOTA-based bifunctional chelators and Sn-precursor of SGMIB, respectively, were evaluated in orthotopic tumor-bearing athymic nude mice. The RCP determined via iTLC of [^225^Ac]-2Rs15d and [^111^In]-2Rs15d were 86.8 ± 2.1% and 91.3 ± 2.1%, respectively. The therapeutic efficacy of [^131^I]-2Rs15d and [^225^Ac]-2Rs15d, as a single agent, were compared to trastuzumab, as well as the combination treatment of [^131^I]-2Rs15d and [^225^Ac]-2Rs15d with trastuzumab. Both [^131^I]-2Rs15d and [^225^Ac]-2Rs15d alone and in combination with trastuzumab showed a significant increase in median survival that remained largely unresponsive to trastuzumab treatment alone. Dosimetry calculations based on ex vivo biodistribution confirmed the accumulation of [^225^Ac]-2Rs15d mainly in tumor and kidneys, however, the absorbed dose is considerably lower than for the ^131^I-labeled Nb. All these results suggest that these radiolabeled Nbs can be valuable vehicles for a theranostic approach to detect and combat HER2 metastatic cancer, as a single agent or as an add-on therapy for treatment-resistant cancers [69].

An overview of HER2 Nb-based radiopharmaceuticals based on different chelator agents and radiometals is provided in Table 3.

The best labeling technique is, therefore, to provide a site-specific conjugation and metabolic stability to avoid cleavage of the radionuclide from the linked protein/Nb. Thus, the choice of the radiolabeling method must be in accordance with the properties of the chosen radionuclide to allow adjustment not only to the labeling of the protein but also to the application required. Still, the technique used must be able to provide good yield, stability and unaltered bioactivity of the product.

**Table 3:** HER2^+^ 2Rs15d Nb-based radiopharmaceuticals using different radiometals and chelating agents.

Entry	Radiometal	Production Mode	Half-Life	Mode of Decay (%)	Maximum Energy (KeV)	Chelator Agent	HER2 Nanobody-Tracer	Phase	Application	Ref.
1	^68^Ga	^68^Ge/^68^Ga generator	1.13 h	*β*^+^ (90)	1899	*p*-SCN-Bn-NOTA	^68^Ga-NOTA-2Rs15d	Phase II ongoing (NCT03331601)	PET	[68,82]
2	^111^In	^111^Cd(p,n)^111^In	2.83 d	EC (100)	245	maleimide-DTPA	^111^In-DTPA-2Rs15d	Preclinical	SPECT	[88]
3						CHX-A″-DTPA	^111^In-DTPA-2Rs15d			[86]
4						*p*-SCN-Bn-CHX-A″-DTPA	^111^In-DTPA-2Rs15d			[87]
5						*p*-SCN-Bn-DOTA	^111^In-DOTA-2Rs15d			[69]
6	^117^Lu	^176^Lu(n,g)^177^Lu	6.71 d	*β^−^* (100)	500	CHX-A″–DTPA	^177^Lu-DTPA-2Rs15d	Preclinical	TRNT	[85]
7						1B4M–DTPA	^177^Lu-DTPA-2Rs15d			
8						*p*-NCS–Bn–DOTA	^177^Lu-DOTA-2Rs15d			
9						DOTA–NHS–ester	^177^Lu-DOTA-2Rs15d			
10						1B4M-DTPA	^177^Lu-DTPA-2Rs15d	Preclinical		[86]
11	^225^Ac	^226^Ra(p,2n)^225^Ac	10 d	*α* (100)	8000	*p*-SCN-Bn-DOTA	^225^Ac-DOTA-2Rs15d	Preclinical	TRNT	[87]
12						*p*-SCN-Bn-CHX-A″-DTPA	^225^Ac-DTPA-2Rs15d	Preclinical		[69]

Ac—Actinium; *β*^+^—positron emission; *β*^−^—beta emission; Cd—Cadmium; d—days; DOTA—1,4,7,10-tetraazacyclododecane-1,4,7,10-tetracetic acid; DTPA—diethylene triamine pentaacetic acid; EC—electron capture; Ga—gallium; Ge—germanium; h—hours; In—indium; Lu—Lutetium; NOTA—1,4,7-triazacyclononane-1,4,7-triacetic acid; PET—positron emission tomography; Ra—Radium; SPECT—single-photon emission computed tomography.

## 5. Preclinical to Clinical Studies: Radiolabeled Nbs as Potential Theranostic Agents

The synthesis and preclinical validation of a novel anti-HER2 Nb—^68^Ga–NOTA–2Rs15d—for immuno-PET was reported by Xavier et al., before first-in-human clinical trials. The probe showed high specific accumulation in the tumor area in ex vivo biodistribution studies and in PET/CT imaging in animals bearing HER2-expressing xenografts. The most remarkable finding of the biodistribution studies was the lower retention of activity in the kidneys after the removal of the His tag from the Nb. Overall, the tracer proved to be safe based on mouse toxicity and dosimetry studies and was considered secure for clinical diagnostic translation [82].

The first clinical trial using ^68^Ga–NOTA–2Rs15d for PET/CT assessment of HER2 was performed by Keyaerts et al. [68]. The study was successfully conducted in twenty breast cancer patients, without the occurrence of adverse effects with administrated activities between 53–174 MBq. The whole-body imaging revealed a favorable biodistribution with the highest accumulation in the kidneys, liver and intestine and low background levels in all the other organs that commonly harbor primary or metastatic BC lesions. Moreover, the blood clearance was rapid, with only 10% of the injected activity remaining in the blood after 1h, allowing the image acquisition at early-times points, within 60–90 min post-injection without the risk of false-positive signals. The tracer elimination occurred mainly through the renal system, similar to the patterns described in rodents, being the urinary bladder wall the organ receiving the highest dose. However, the average radiation burden was considered acceptable for diagnosis for the maximum administered activity. Although not being the primary objective of this study, the authors also evaluated the uptake in primary and metastatic lesions, with uptakes ranging between 0.7 and 11.8 for primary tumors and a clear tracer accumulation in at least one metastatic lesion with a SUV ranging from 3.1 to 6.0 between 60- and 90-min post-injection, being the later proposed the optimal imaging time-point to obtain images with an optimal signal-to-noise ratio. This first in-human use of radiolabeled Nb demonstrated the advantages over the slow blood clearance of the full therapeutic antibody resulting in late imaging time-points of 2 or 3 days and a high radiation burden for patients [68].

A phase II trial is currently ongoing to investigate the uptake of ^68^Ga-anti-HER2 VHH1 in HER2^+^ BC brain metastases using PET/CT imaging (NCT03331601) [96]. If successful, the same approach could support the development of therapeutic analogs for TRNT.

Another promising radiolabeled Nb for TRNT is the anti-HER2 sdAb 2Rs15d labelled with the radiohalogen ^131^I using [^131^I]SGMIB. Preclinical studies in two distinct HER2^+^ murine xenograft models revealed a high and specific tumor uptake and a more favorable biodistribution profile with a faster clearance from kidneys as compared to ^68^Ga–NOTA–2Rs15d. The high contrast micro SPECT/CT images showed the highest tumor uptake at 1h post-injection with a value of 20% that was retained for at least 24h. These favorable characteristics combined with the theranostic potential of ^131^I encouraged the use of this compound as a potential TRNT agent in metastatic patients with HER2^+^ BC [78].

In 2020, it was reported a phase I trial of this new promising candidate aimed at evaluating the safety, biodistribution, radiation dosimetry and tumor imaging potential in healthy volunteers and BC patients. The study encompassed six healthy volunteers and three patients with metastatic HER2^+^ BC to assess the uptake of ^131^I-GMIB-Anti-HER2-VHH1 in metastatic lesions. No drug adverse related effects were observed in healthy volunteers or patients after intravenous administration of low activities ^131^I-GMIB-anti-HER2 (~40 MBq). The biological half-life was about 8 h and the unbound radiotracer was primarily eliminated via the kidneys. Still, the SPECT/CT imaging of patients with advanced HER2^+^ BC showed accumulation of ^131^I-GMIB-Anti-HER2 Nb in metastatic lesions. Based on dosimetry analysis, kidneys are the dose-limiting organs upon dose escalation, but kidney toxicity should only occur at very high administered activities [97]. A dose escalation is planned in a subsequent phase I/II to assess the therapeutic window of ^131^I-GMIB-Anti-HER2 (NCT04467515).

The same 2Rs15d Nb fragment was radiolabeled with ^99m^Tc by Vaneycken et al. in 2011. The radiolabelled compound revealed good stability at least up to 24 h in PBS and serum and high specificity for the HER2 antigen. SPECT imaging studies in two HER2^+^ mouse models showed a fast blood clearance, low accumulation in nontarget organs except kidneys and high tumor-to-blood and tumor-to-muscle ratios at 1 h after intravenous injection [83].

More recently in 2021, Zhao and co-workers developed a novel ^99m^Tc-labelled anti-HER2 sdAb designed as ^99m^Tc-NM-02. The first in-human studies were performed in 10 patients with BC (NCT04040686) to assess safety, radiation dosimetry, biodistributions and tumor targeting potential. No-drug related adverse effects were registered and the SPECT/CT images demonstrated favorable biodistribution, with a preferential accumulation in the liver and kidney and mild uptake in the spleen, intestines and thyroid. The maximal standard uptake in primary lesions and regional metastasis were observed at 1 and 2 h post-injection with a range of levels of SUV_max_ of 0.35–11.18 and considered the SUV_max_ of 1.5 as a reasonable cutoff value for determining HER2 positivity. Larger clinical trials are being planned by the authors not only for noninvasive detection of HER2 expression but also for targeted radionuclide therapy in BC [84].

The first-in human applications of the anti-HER2 Nb referred to as 2Rs15d, present high affinity and in vivo tumor targeting to a HER2 epitope distinct from those targeted by trastuzumab and pertuzumab. This noncompeting character allows their administration to patients undergoing anti-HER-2 targeted therapy who eventually progress and might represent an effective new therapeutic option.

## 6. Conclusions

Nbs are increasingly being used as targeting molecular tools for imaging and/or TRNT due to their unique characteristics that allow high contrast imaging at early time points after administration and effective therapy with minimal nonspecific toxicity. Here, we describe the various radiolabeling strategies used to incorporate radionuclides into Nbs for SPECT and PET applications. Several methods were discussed including direct radiolabeling, indirect radiolabeling via prosthetic group and indirect radiolabeling via complexation. To facilitate the clinical translation, is it important to ensure that the radiolabeling process has no impact on the affinity and that the Nbs are homogenously labeled. Furthermore, the development of prosthetic groups and chelators, with improved effects on the pharmacokinetics of Nbs is of particular interest, especially considering kidney retention, a critical point for potential toxicity, particularly in the therapeutic setting. Owing to the preclinical data obtained so far, the utility of these Nb tracers as theranostic agents has been broadly recognized. However, the translation to the clinic is still very limited, which may be due to the limiting factors as toxicity studies and the expensive and time-consuming process to translate to good manufacturing practice (GMP) production.

## Figures and Tables

**Table 1:** Main characteristics of different engineered antibody fragments.

	Molecular Weight (kDa)	Avidity	Clearance Route	Serum Half-Life	Optimal Time for Imaging	Ref.
IgG	150	Bivalent	Liver	110 h	4–7 d	[41,47]
F(ab)’2	110	Bivalent	Liver/Kidney	9 h	72–120 h	[41]
Minibody	80	Bivalent	Liver	5–12 h	24–48 h	[47]
Diabody	50	Bivalent	Kidney	2–5 h	12–24 h	[47]
Fab	55	Monovalent	Kidney	28 min	2–5 h	[41]
scFv	25	Monovalent	Kidney	10 min	1–3 h	[41]
Nb	15	Monovalent	Kidney	1–1.5 h	1–1.5 h	[41]
Affibody	6–7	Not applicable	Kidney	4–14 min	1–2 h	[41,47,48,49]

**Table 2:** A brief overview of the radiolabeling strategies including radioisotope, nanobody, labeling conditions, radiochemical yield and radiochemical purity.

Entry	Radiolabeling Method	Nb	Isotope	Intermediate	Radiolabeling Conditions	RCY (%)	RCP (%)	Ref.
1	Direct	5F7GGC	^125^I	Directly	r.t., 10 min	83.6 ± 5.0	>99	[74]
2				Directly	r.t., 10 min	86.2 ± 1.6	>98	[75]
3	Indirect via prosthetic group	5F7		[*I]SGMIB	-	-	-	[76]
4		5F7GGC	^131^I	[*I]IB-Mal-D-GEEEK	r.t., 45 min	91.2 ± 4.4	>99	[74]
5		5F7GGC		[*I]IB-Mal-D-GEEEK	r.t., 45 min	69.6 ± 5.6	>98	[75]
6		5F7GGC		[*I]SGMIB	r.t., 10 min	50.4 ± 3.6	>98	
7		5F7		[*I]SGMIB	-	28.9 ± 13.0	98.4	[77]
8				*iso*-[*I]SGMIB	-	33.1 ± 7.1	98.6	
9		2Rs15d		[*I]SGMIB	20 °C, 30 min	36.5 ± 12.8	>97	[78]
10		5F7GGC		[*I]MEGMIB	37 °C, 45 min	45 ± 7	>99	[79]
11		5F7	^211^At	SAGMB	20 °C, 20 min	38.4 ± 15.6	97.8	[77]
12				*iso*-SAGMB	20 °C, 20 min	39.5 ± 6.8	98.9	
13		2Rs15d	^18^F	[^18^F]SFB	r.t., 20 min	5–15	>97	[80]
14		5F7		^18^F-RL-1	20 °C, 20 min	-	>95	[81]
15		5F7		^18^F-RL-1	20 °C, 20 min	-	95	[76]
16				[^18^F]SFB	-	-	95	
17	Indirect via complexation	2Rs15d	^68^Ga	*p*-SCN-Bn-NOTA	r.t., 5 min	>97	99	[82]
18		2Rs15d-His6			r.t., 5 min	>97	99	
19		2Rs15d	^99m^Tc	His tag	50 °C, 90 min	-	>99	[83]
20		NM-02		His tag	50 °C, 60 min	-	97.7 ± 1.2	[84]
21		2Rs15d-His	^177^Lu	CHX-A”-DTPA	r.t., 30 min	-	91 ± 1.06	[85]
22		2Rs15d-His		1B4M-DTPA	r.t., 30 min	-	94 ± 2.21	
23		2Rs15d-His		*p*-NCS-Bn-DOTA	50 °C, 45 min	-	96 ± 2.10	
24		2Rs15d-His		DOTA-NHS-ester	50 °C, 45 min	-	96 ± 1.97	
25		2Rs15d		1B4M-DTPA	r.t., 30 min	97.2 ± 2.5	>99	[86]
26		2Rs15d	^225^Ac	*p*-SCN-Bn-DOTA	50 °C, 90 min	>90	>95	[87]
27		2Rs15d		*p*-SCN-Bn-DOTA	55 °C, 90 min	-	86.8 ± 2.1	[69]
28		2Rs15d	^111^In	*p*-SCN-Bn-CHX-A”-DTPA	55 °C, 30 min	-	91.3 ± 2.1	
29		2Rs15d		Malemide-DTPA	50 °C, 30 min	-	94.0 ± 4.9	[88]

DOTA—1,4,7,10-tetraazacyclododecane-1,4,7,10-tetracetic acid; DTPA—diethylene triamine pentaacetic acid; NOTA—1,4,7-triazacyclononane-1,4,7-triacetic acid; r.t.—room temperature; RCP—radiochemical purity; RCY—radiochemical yield.
